# The Relationship between Exercise and Mental Health Outcomes during the COVID-19 Pandemic: From the Perspective of Hope

**DOI:** 10.3390/ijerph19074090

**Published:** 2022-03-30

**Authors:** Yingying Yao, Jianqiao Chen, Dan Dong, Yi Feng, Zhihong Qiao

**Affiliations:** 1 Faculty of Psychology, Beijing Normal University, Beijing 100875, China; yaoyingying@xmu.edu.cn (Y.Y.); dongdan92@outlook.com (D.D.); 2 Counseling and Education Center, Xiamen University, Xiamen 361005, China; 3 Mental Health and Education Center, University of International Business and Economics, Beijing 100029, China; chenjq@uibe.edu.cn; 4 Mental Health Center, Central University of Finance and Economics, Beijing 100081, China

**Keywords:** exercise, hope, mental health outcomes, COVID-19, health promotion

## Abstract

The unexpected outbreak of COVID-19 triggered fear and anxiety in the general population. Exercise was one of the most widely promoted methods to improve body function when socially restricted. This study aims to examine the role of exercise in relieving stressful mental health outcomes (anxiety and depressive symptoms) during the COVID-19 pandemic and explore the underlying mechanism from the perspective of hope, using a combination of goal-directed planning (pathways) and motivation (agency). A cross-sectional online survey recruiting 2390 Chinese participants was conducted during the COVID-19 pandemic in China. A series of questions and scales, including the self-designed exercise questionnaire, the Adult Dispositional Hope Scale, the Generalized Anxiety Disorder Scale-7 and the Patient Health Questionnaire-9, were used to measure exercise, hope, anxiety symptoms and depressive symptoms, respectively. A structural equation model was constructed to test the hypothesis that exercise benefits mental health outcomes through the mediating role of hope. Our results showed that exercise relieved stressful mental health outcomes via three paths: one direct path (β = −0.077, 95% CI = (−0.138, −0.017), *p* < 0.01), one indirect path through hope of pathways thinking (β = −0.046, 95% CI = (−0.064, −0.027), *p* < 0.001) and another indirect path through hope of agency thinking (β = −0.060, 95% CI = (−0.081, −0.039), *p* < 0.001). Our results showed that exercise could alleviate stressful mental health outcomes by promoting both hope of pathway thinking and agency thinking. It provided practical insights into psychological prevention and intervention by means of exercise during the COVID-19 pandemic.

## 1. Introduction

### 1.1. Background 

Over 467 million people were infected by the coronavirus disease 2019 (COVID-19) worldwide, with over 6.05 million fatalities as of March 2022 [1]. The unexpected outbreak of COVID-19, especially considering its high transmission rate, undoubtedly triggered fear and anxiety in the general population [2]. However, not everyone responded in the same manner. Understanding how the general population reacted to the disease outbreak and identifying coping strategies to help adjust to life challenges brought by the pandemic are urgent and important topics for health psychology.

Several studies have investigated the mental health outcomes of individuals during the pandemic, and protective factors to improve mental health have been increasingly reported [3,4]. Exercise, one of the most widely promoted methods and meaningful prevention factors of symptoms related to the COVID-19 pandemic [5], is also a protective factor against the emergence of stressful mental health outcomes [6].

### 1.2. Exercise and Mental Health

According to the cross-stressor adaptation hypothesis, regular exercise facilitates adaptations in individuals’ stress response systems and then ameliorates physiological sufferings imposed by psychological stressors [7]. Meanwhile, numerous studies have reported that regular exercise contributes not only to better physical health but also to improved mental health outcomes [3,4,8]. People with high levels of physical activity generally have a lower risk of depression than those with low levels [9]. Exercise participation protects individuals from anxiety-related symptoms and disorders [10]. Exercise engagement affects mental health during social distancing due to the COVID-19 pandemic, and moderate-to-vigorous daily exercise is negatively related to poor mental health [11]. Those engaging in moderate-to-vigorous physical activity have lower odds of prevalent anxiety and depressive symptoms [12]. Conversely, higher physical inactivity is associated with more anxiety and depressive symptoms during the COVID-19 pandemic [13]. The biochemical and physiological changes of exercise, such as increases in neurogenesis and reductions in inflammatory and oxidant markers [14], have also been proven to benefit mental health outcomes. Therefore, we assumed that the amount of exercise is negatively associated with affective symptoms during this pandemic.

### 1.3. Exercise and Hope

To our knowledge, how exercise benefits one’s mental health during the pandemic remains unclear. Better physical fitness generally suggests a lower probability of infection or faster recovery, which relieves pressure and potentially mobilises agency and resources. According to the hope theory, hope is defined as the perceived capability to derive pathways to desired goals and motivate oneself via agency thinking to use those pathways [15]. Hope has been proven to play an integral role in promoting health in health programmes [16] and in coping with post-traumatic stress [17,18]. Thus, believing that hope-filled attitudes can also help coping with and adapting to this pandemic is reasonable.

During the current COVID-19 pandemic, exercise participation is expected to relieve physiological symptoms, such as chest distress, panting, and other typical symptoms of anxiety. The direct benefits reinforce individual’s actions and motivate further exercise plans, known as the positive-feedback loop. Therefore, exercise and the following positive feedback give rise to subsequent goals and enhance pathways thinking. As goal setting is an important part of pathways thinking, exercise is assumed to improve pathways thinking.

When individuals are willing to adopt exercise as a coping method for stress generated by COVID-19 risk, it leads to a higher degree of autonomy and more inherent tendencies. According to self-determination theory [19] and empirical research results [20,21], the more autonomy is gained, the more effective and lasting the change will be. Therefore, the more exercise one engages in, the more autonomous and motivated one will become, which is the second facet of hope–agency thinking. To sum up, exercise was assumed to facilitate the two facets of hope.

### 1.4. Hope and Mental Health

Hope is described as the “rainbow in the mind” and “light in the darkness”; hope empowers individuals to find meaning and supports them through difficulties [22]. As a health-promoting psychological strength, hope has greater potential than other general protective factors as it is an enduring and specific-goal-directed process [23]. The ability to set and adjust one’s goals, to continually solve problems and make effort to go beyond barriers, may help to relieve stress and reduce negative reactions [24]. Several studies have supported the assertion that hope is associated with fewer negative mental health outcomes (e.g., depressive symptoms, anxious symptoms, and suicidal risks) [25,26]. Hope that is relevant to a specific goal can support individuals through ongoing stressful emotions and bitterness and help make meaning out of their sufferings. This psychological power adds to one’s psychological defences [27], thereby protecting from enduring psychological distress in traumatic situations [28] and promoting positive outcomes after trauma [29].

### 1.5. Exercise, Hope, and Mental Health

Hope may be an important variable to understand the association between exercise and mental health outcomes. A case study of exercise among seven individuals with chronic obstructive pulmonary disease found that hope persists despite the condition difficulties [16]. Some exercise intervention programmes, called HOPE (e.g., Home-based Older People’s Exercise, Hormones and Physical Exercise), imply hope as the goal, yet none of them have explicitly adopted hope as a psychological construct [30,31]. 

Limited studies that measured hope revealed a significant correlation between a self-reported rise in exercise and hope for Latino populations [32] and for undergraduate students [21]. As previously mentioned, exercise is one of the most widely promoted methods to improve body function when socially restricted, and exercise may also benefit the two types of hope thinking. Therefore, exercise is hypothesised to protect against poor mental health outcomes via hope.

### 1.6. Present Study 

This study aimed to examine a conceptual model to investigate the association between exercise and mental health outcomes and test the mediating effect of hope in a cross-sectional sample of Chinese individuals during the COVID-19 pandemic.

The study proposed a mediation model, with the following hypotheses. First, exercise will negatively predict mental health outcomes (including anxiety and depressive symptoms) during the COVID-19 pandemic. Second, exercise will significantly predict the two components of hope, namely pathways thinking and agency thinking. Third, both pathways and agency thinking will mediate the relationship between exercise and mental health outcomes.

## 2. Materials and Methods

### 2.1. Participants and Sampling

In this cross-sectional study, an online survey was conducted among Chinese individuals from 10 April to 29 April 2020. During that period, students and staff were not allowed to return to campus and socially isolated at home. Mixed methods of convenience sampling and snowball sampling were used to collect data by distributing questionnaire links to the participants in three Chinese universities. Inclusion criteria were as follows: (1) participants were able to understand the Chinese language; (2) neither participants nor their family members were infected with COVID-19; (3) the participants were not diagnosed with mental illness before the pandemic, not limited to anxiety and depression disorders. A total of 2390 participants were included in the final sample. 

Prior to investigation, the participants were informed of the purpose and procedure of the study by a notification sent through the WeChat App. Online informed written consent was obtained from all participants at the beginning of the questionnaire. This study was approved by the Research Ethics Review Committee of Beijing Normal University.

### 2.2. Measures

***Exercise.*** The following three items were used to measure the level of exercise: (1) “Do you have a habit of exercising during the pandemic”? on a four-point Likert scale 1: not at all, 2: occasionally, 3: at least two low-intensity exercises per week, 4: at least two high-intensity exercises per week; (2) “Average duration (hours) of daily exercise in the past 2 weeks”; (3) “Average number of daily exercises in the past 2 weeks”. The latter two items required the participants to report the number of hours and times of daily exercises. Physical exercise as a latent variable was constructed on the above three items (Cronbach’s *α* = 0.573). 

***Hope.*** Hope was assessed using the 12-item Adult Dispositional Hope Scale (ADHS) [33]. The ADHS consists of two subscales: pathways thinking and agency thinking. Each subscale was calculated by summing up four items, for example, “there are lots of ways around any problem” for pathways thinking and “energetically pursue my goals” for agency thinking. The remaining four items were fillers. Respondents rated how accurately each item described them on a seven-point Likert-type scale, ranging from 1 (definitely false) to 7 (definitely true). Higher scores reflect a higher degree of hope. In the present sample, the internal consistency of ADHS was good (Cronbach’s *α* = 0.872).

***Mental health outcomes.*** The latent variable “mental health outcomes” was the overall mental health outcome variable constructed on anxiety and depressive symptoms, for the reason that anxiety and depression diagnoses frequently tend to co-occur and that their symptoms are highly correlated [34]. The seven-item Generalized Anxiety Disorder Scale was used to measure the severity of generalized anxiety disorder. Participants rated the frequency of symptoms over the past 2 weeks on a four-point scale from 0 (not at all) to 3 (nearly every day). An anxiety score was calculated by summing up the seven items (Cronbach’s *α* = 0.929), with a higher score indicating a greater severity of anxiety symptoms. Anxiety symptoms were identified based on a cut-off score of 5 in this study [35]. The Patient Health Questionnaire, a nine-item self-report measure, was used to assess the frequency of the occurrence of depressive symptoms over the past two weeks on a four-point scale from 0 (not at all) to 3 (nearly every day). A total score of depressive symptoms was created (Cronbach’s *α* = 0.911) by summing up nine items, with higher scores indicating a greater severity of depressive symptom. The cut-off used for the depressive symptoms was 5 in this study [36].

### 2.3. Data Analysis

Descriptive statistics were initially reported on demographic characteristics and the variables of interest. Comparisons were made between the participants with and those without anxiety symptoms and between participants with and those without depressive symptoms. A Mann–Whitney U test was performed for comparing age, which was not normally distributed, and a chi-square test was conducted for comparing sex and ethnicity. Correlation analyses were performed to examine the bivariate associations among the variables of interest. Spearman correlations was used to analyse all continuous variables that were not normally distributed.

The hypothesised mediating effects were examined using structural equation modelling (SEM). To assess the model fit, we used normed *χ*^2^, Comparative Fit Index (CFI; 0.90 or above indicating an acceptable fit), Tucker–Lewis Index (TLI; 0.90 or above reflecting an acceptable fit), standardised root mean residual (SRMR; 0.08 or below reflecting an acceptable fit), and root mean square error of approximation (RMSEA; 0.08 or below reflecting an acceptable fit) as indicators of model fitness [37]. Additionally, the 95% bias-corrected confidence interval (CI) from 5000 resamples was generated by a bias-corrected bootstrapping method to examine the significance of the moderated mediation effect. All the above analyses were performed using SPSS version 23.0 and Mplus version 8.0. The two-sided significance level was set at 0.05.

## 3. Results

### 3.1. Background Characteristics and Covariates

The final sample consisted of 2390 participants, of whom 68.91% were women. Table 1 shows the background characteristics of the participants. The mean age was 23.70 (SD = 8.80) years, ranging from 16 to 64 years. Most participants were Han Chinese (92.84%). No significant differences were found between participants with and without affective disorders on demographic characteristics (e.g., sex, ethnicity, or age). Nearly a third of the participants reported significant symptoms of depression and anxiety during the pandemic, and 650 participants (27.2%) reported depression as well as anxiety symptoms. 

Table 2 shows the correlation matrix among all the variables included in the model. The Spearman’s correlation test was used to analyse the correlation between exercise, pathways thinking of hope, agency thinking of hope, anxiety symptoms, and depressive symptoms. A significant positive correlation was found between anxiety and depressive symptoms (*r* = 0.72, *p* *<* 0.001). Anxiety symptoms were significantly associated with exercise (*r* = −0.06, *p* *<* 0.001), pathways thinking (*r* = −0.25, *p* *<* 0.001), and agency thinking (*r* = −0.21, *p* *<* 0.001). Significant correlations were noted between depressive symptoms and exercise (*r* = −0.11, *p* *<* 0.001), pathways thinking (*r* = −0.33, *p* *<* 0.001), and agency thinking (*r* = −0.34, *p* *<* 0.001).

### 3.2. Mediating Effects of Hope

We tested the model containing the latent variable exercise as the predictor, the latent variable mental health outcomes as the dependent, and sex, age, and ethnicity as covariates in the SEM. The SEM model fit the data well (*χ*^2^/*df* = 6.71, CFI = 0.971, TLI = 0.952, RMSEA = 0.049, SRMR = 0.031). We found a significant main effect of physical exercise on mental health outcomes (*β* = −0.084, 95% CI = (−0.131, −0.072), *p* *<* 0.001). The results showed that the more exercise one takes, the less stressful mental health outcomes one experiences. Thus, hypothesis 1 was verified.

**Figure 1:**
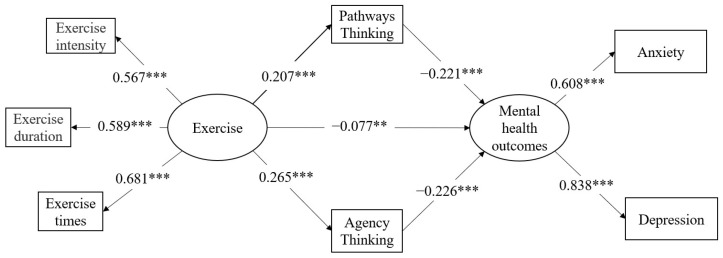
Model of indirect effect of exercise on mental health outcomes through pathways thinking and agency thinking. ** *p* < 0.01, *** *p* < 0.001.

We conducted an SEM to test our mediation hypothesis with exercise as the latent predictor, and hope (pathways thinking and agency thinking) as the mediator on the mental health outcomes, which is the latent outcome variable. Sex, age, and ethnicity were controlled as covariates in the SEM. Figure 1 shows the final SEM model, which fit the data well (*χ*^2^/*df* = 9.923, CFI = 0.951, TLI = 0.924, RMSEA = 0.061, SRMR = 0.041).

The operating results of the theoretical model showed that after controlling the covariates, exercise had a significant positive effect on pathways thinking (β = 0.207, 95% CI = (0.164, 0.251), *p* < 0.001) and agency thinking (β = 0.265, 95% CI = (0.219, 0307), *p* < 0.001). Pathways thinking (β = −0.221, 95% CI = (−0.233, −0.126), *p* < 0.001) and agency thinking (β = −0.226, 95% CI = (−0.241, −0.147), *p* < 0.001) negatively predicted mental health outcomes significantly. Thus, hypothesis 2 was supported.

The mediating role of hope was also detected in the hypothesised model. As indicated in the results, more exercise predicted a better affective state via three paths as follows: one direct path (*β* = −0.077, 95% CI = (−0.138, −0.017), *p* < 0.01), one indirect path through pathways thinking (*β* = −0.046, 95% CI = (−0.064, −0.027), *p* *<* 0.001), and another indirect path through agency thinking (*β* = −0.060, 95% CI = (−0.081, −0.039), *p* *<* 0.001). Exercise was proven to significantly improve mental health outcomes, and hope (pathways thinking and agency thinking, specifically) partially mediated this process. Thus, hypothesis 3 was confirmed.

## 4. Discussion

This cross-sectional study is among the few that attempted to examine the relationship between exercise and hope on mental health outcomes during the COVID-19 pandemic. There were three main findings in this study.

Firstly, exercise was verified to negatively correlate with stressful mental health outcomes (anxiety and depressive symptoms) during the COVID-19 social isolation period. This provides further evidence to previous literature [11] during the pandemic. Exercise, providing positive responses and adaptations, might affect the circulatory levels of two neurotransmitters (serotonin and dopamine), upregulate the BDNF-Serotonin systems [38], and decrease anxiety and depression. Additionally, there were several plausible explanations of the beneficial effects of physical exercise include reductions in inflammatory and oxidant markers [14,39], which are activated during mental disorders, improvement of self-efficacy [40], as well as enhanced hope, the second finding in the study.

Secondly, the associations between exercise and the two dimensions of hope were explored. Exercise positively affected both pathways thinking and agency thinking, indicating that exercise contributes to enhanced hope levels, which is consistent with the results from previous investigations [21,32]. Our study showed that an individual’s hopeful thinking, both pathways thinking and agency thinking, could be positively facilitated by exercise. Exercise is one of the most widely promoted methods and has been proved to reduce the symptoms related with the COVID-19 pandemic [5]. Performing exercise enhances one’s sense of power and self-agency to believe that one can take active actions and reinforce self-esteem [6] to fend off the potential threats brought by the pandemic, rather than running out of solutions and passively waiting.

Thirdly, the results supported that hope played an important mediating role in the relationship between exercise and affective symptoms. In this study, both pathways and agency thinking mediated the relationship between exercise and mental health outcomes. Exercise not only gives rise to the agency necessary to overcome difficulties, but also provides an effective approach to fight against these difficulties. Hope (both pathways and agency thinking), personal goal-based beliefs in shaping outcome expectations, acts as a protective factor against maladaptive mental outcomes [41]. High levels of hope facilitate realistic goals and melt away hopelessness, which adversely affects people’s confidence. Consequently, they are more resilient against negative events. The virtuous circle of “more exercise-higher control-more hopeful-more exercise/action” generates internal power and resource, which eventually reduces the negative effect of the pandemic on one’s mental health.

One major theoretical contribution of our study is that it confirmed the function of hope to affective health and clarified the two dimensions’ separate effects on affective symptoms, which enriches the theoretical framework of hope and adds empirical evidence to the hope theory during the COVID-19 pandemic. This offers a natural research opportunity and contributes to the hope theory in a specific period and social circumstance. Another theoretical implication concerns the relationship between exercise and hope. We found that exercise has a positive impact on hope. Since no previous research has been conducted on the effect of exercise on hope, this study is novel and provides an explanation of how exercise could improve mental health during the pandemic.

Although the cultural ramifications of the physical restrictions remain unclear, this study showed how exercise under physical restrictions affects the general population and their mental health/illness. The present findings suggest that engaging in exercise during the COVID-19 self-isolation period could be one of the most effective prevention factors for people with affective symptoms. These findings are consistent with previous studies conducted during non-pandemic times [9,10,42]. Exercise is one of the effective rehabilitation methods for patients with mild coronavirus pneumonia, and personalised remote-guided exercise rehabilitation has been proven to effectively alleviate patients’ symptoms [43]. Exercise helps the general population to improve immunity by enhancing physical fitness. On a psychological level, exercise helps improve an individual’s resilience [44]. During the period of pandemic prevention and control, individuals who exercise are more likely to enhance their efficacy of controlling emotions or symptoms. The belief that “I can do something to deal with the risk” inspires self-control and self-management and increases the hope to overcome the challenge. 

Similar to previous research, our results also imply that hope, which can be inculcated, may promote well-being and ameliorate affective symptoms in individuals affected by COVID-19. To enhance hope, brief interventions, particularly strategies from motivational interviewing and more simplistic goal identification and attainment exercises, could be implemented in mental health nursing services [45]. Both pathways and agency thinking have been verified as efficient buffers for anxiety or depressive disorders. Thus, the promotion of pathways thinking (such as making reasonable exercise plans; setting more specific, evaluable, and achievable goals based on an individual’s physical conditions and personal fitness during the isolation period; improving one’s scientific understanding of diseases; and implementing protection measures to reduce risks) as well as the evaluation of agency thinking (such as improving the consistency in exercising habits by self-encouragement, strengthening exercise confidence by progressing with small steps, and searching for social support to confront anxiety and depressive symptoms) should be considered in future psychological inventions. Goal-motivated thoughts and behaviours may support individuals to overcome otherwise frustrating barriers and experiences, regain self-control, and enhance self-confidence, thereby reducing mental health costs. 

This study has several limitations. Firstly, its cross-sectional design cannot rule out the possibility of bi-directionality, which precludes an examination of causality. Future longitudinal studies should be conducted to further examine the effect of exercise. Secondly, only the amount and frequency of exercise were considered in this study, because social isolation might notably limit the amount of exercise despite the fact that the Chinese government did not prohibit outdoor exercise. Ingram suggested that changes in exercise levels were also important for mental health [46]. Therefore, how the amount and change of exercise levels interact to influence mental health will be a consideration in future studies. Thirdly, most of the participants were college students, which may have caused selection bias in the representativeness and limit the generalization of the results. The implications of the current conclusions to other groups requires further inquiry. Finally, the self-reported method of exercise in the study was a compromised under the pandemic situation. If conditions permit, both objective assessments, such as field observation or real-time recording, and subjective assessments can be comprehensively considered to better reflect the level of exercise.

There are several research directions that can be pursued in the future. Firstly, this study used exercise as the antecedent of the model. Future studies could extend the model one step ahead by examining what precedes exercise and shed light on the exercise promotion/intervention programs. More refined research about which types of exercise or what intensity of exercise could be most effective in promoting mental health are also valuable topics. Secondly, future research should explore other possible mediators, such as self-efficacy and aloneness, as well as the hope to broaden the view of the mediation mechanism. Additionally, psychosocial factors that could influence mental health outcomes could be included in the future. For example, social support, especially support from family members, may be one crucial factor for the public, especially when socially isolated at home. Thirdly, future studies could also explore the implication of domain-specific exercise, e.g., Virtual Reality exercise, which has been reported to facilitate improved cognition and psychological outcomes [47]. Exercise, similar to eating and sleeping, not only enhances immune function but also reduces psychological pressure and tension, which is of great significance in establishing preventive protocols. However, these findings also imply that those with physical inactivity during the COVID-19 social distancing period may need extra support to enhance their mental health status.

## 5. Conclusions

This study demonstrated the protective effects of exercise and the underlying mechanism of exercise on mental health outcomes during the COVID-19 pandemic. Our results showed that exercise has direct immune-boosting benefits and indirect benefits through the mediating effects of hope for mental disorders. It is suggested that exercise may be a valuable prevention method, not only for physical health but also for mental health, by helping individuals enhance their hope in the pandemic.

## Figures and Tables

**Table 1:** Demographic characteristics of the study sample (*n* = 2390).

		With Depressive Symptoms (*n* = 935) *n* (%)	Without Depressive Symptoms (*n* = 1455) *n* (%)	*p* Value	With Anxiety Symptoms (*n* = 780) *n* (%)	Without Anxiety Symptoms (*n* = 1610) *n* (%)	*p* Value
Sex	Male (*n* = 743)	302 (32.3%)	441 (30.3%)	0.319	225 (28.9%)	518 (32.2%)	0.109
	Female (*n* = 1647)	633 (67.7%)	1014 (69.7%)		555 (71.2%)	1092 (67.8%)	
Ethnic group	Han (*n* = 2219)	868 (92.8%)	1351 (92.9%)	0.987	720 (92.3%)	1499 (93.1%)	0.499
	Others (*n* = 171)	67 (7.2%)	104 (7.2%)		60 (7.7%)	111 (6.9%)	
Mean Age (SD)		22.82 ± 7.63	4.26 ± 9.44	0.151	23.58 ± 8.34	23.75 ± 9.02	0.088

Note. The cut-off score for participants with and without anxiety or depressive symptoms is 5 in this study, using the scales of the GAD-7 and the PHQ-9 respectively. *p* value: Chi-square test for sex and ethnic comparisons; Mann–Whitney U test for age comparisons.

**Table 2:** Correlation between main variables (*n* = 2390).

	1	2	3	4	5	6	7
1 Sex	1.00						
2 Age	0.06 **	1.00					
3 Ethnic group	0.06 **	0.03	1.00				
4 Exercise	−0.10 **	−0.09 **	0.01	1.00			
5 Pathways thinking	−0.03	0.07 **	0.02	0.15 **	1.00		
6 Agency thinking	−0.03	0.15 **	0.01	0.20 **	0.63 **	1.00	
7 Anxiety	0.04 *	0.05 *	0.03	−0.06 **	−0.25 **	−0.21 **	1.00
8 Depression	−0.02	−0.04	0.01	−0.11 **	−0.33 **	−0.34 **	0.72 **

*Note*. The sex was coded as dummy variable “0 = male, 1 = female”; Ethic group was coded as dummy variable “0 = Han ethnic, 1 = others”. * *p* < 0.05, ** *p* < 0.01.

## Data Availability

The data that support the findings of this study are available on request from the corresponding author Y.F. The data are not publicly available due to their containing information that could compromise the privacy of research participants.
